# Biological Evaluation and Molecular Docking with In Silico Physicochemical, Pharmacokinetic and Toxicity Prediction of Pyrazolo[1,5-*a*]pyrimidines

**DOI:** 10.3390/molecules25061431

**Published:** 2020-03-21

**Authors:** Ahmed M. Naglah, Ahmed A. Askar, Ashraf S. Hassan, Tamer K. Khatab, Mohamed A. Al-Omar, Mashooq A. Bhat

**Affiliations:** 1 Department of Pharmaceutical Chemistry, Drug Exploration and Development Chair (DEDC), College of Pharmacy, King Saud University, Riyadh 11451, Saudi Arabia; anaglah@ksu.edu.sa (A.M.N.); malomar1@ksu.edu.sa (M.A.A.-O.); 2 Peptide Chemistry Department, National Research Centre, Dokki, Cairo 12622, Egypt; 3 Botany and Microbiology Department, Faculty of Science (Boys), Al-Azhar University, Cairo, Egypt; 4 Organometallic and Organometalloid Chemistry Department, National Research Centre, Dokki, Cairo 12622, Egypt; tamer_khatab@hotmail.com; 5 Department of Pharmaceutical Chemistry, College of Pharmacy, King Saud University, Riyadh 11451, Saudi Arabia; mabhat@ksu.edu.sa

**Keywords:** pyrazolo[1,5-*a*]pyrimidine, antimicrobial, immunomodulatory, Lipinski’s rule, molecular docking, enzyme inhibitor

## Abstract

Pyrazolo[1,5-*a*]pyrimidines **5a**–**c**, **9a**–**c** and **13a**–**i** were synthesized for evaluation of their in vitro antimicrobial properties against some microorganisms and their immunomodulatory activity. The biological activities of pyrazolo[1,5-*a*]pyrimidines showed that the pyrazolo[1,5-*a*]pyrimidines (**5c**, **9a**, **9c**, **13a**, **13c**, **13d**, **13e** and **13h**) displayed promising antimicrobial and immunomodulatory activities. Studying the in silico predicted physicochemical, pharmacokinetic, ADMET and drug-likeness properties for the pyrazolo[1,5-*a*]pyrimidines **5a**–**c**, **9a**–**c** and **13a**–**i** confirmed that most of the compounds (i) were within the range set by Lipinski’s rule of five, (ii) show higher gastrointestinal absorption and inhibition of some CYP isoforms, and (iii) have a carcinogenicity test that was predicted as negative and hERG test that presented medium risk. Moreover, the molecular docking study demonstrated that the compounds **5c**, **9a**, **9c**, **13a**, **13c**, **13d**, **13e** and **13h** are potent inhibitors of 14-alpha demethylase, transpeptidase and alkaline phosphatase enzymes. This study could be valuable in the discovery of a new series of drugs.

## 1. Introduction

Treatment of infectious diseases remains a worldwide problem because of the increasing multi-drug resistance caused by human pathogenic microbes. Therefore, the design of new compounds acting as antimicrobial agents is an essential approach to overcome the problem of drug resistance [1].

**Figure 1:**
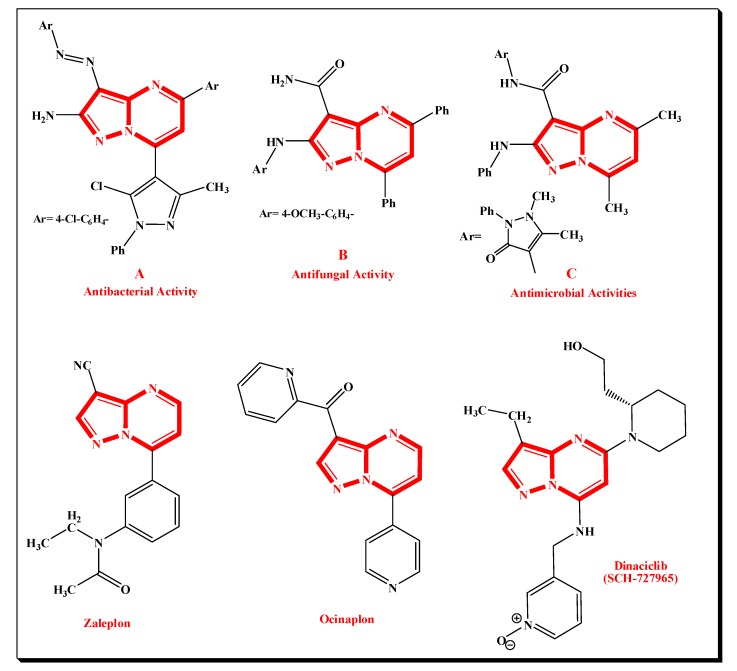
Examples of biological activities of pyrazolo[1,5-*a*]pyrimidines and the structures of some drugs.

In recent years, pyrazolo [1,5-*a*]pyrimidine derivatives have received a special interest due to their diverse biological and pharmacological activities including DNA binding and anti-tubercular, antioxidant, antibacterial and anticancer activities [2,3,4,5,6,7,8]. Among these pyrazolo[1,5-*a*]pyrimidine derivatives, compound **A** showed potent antibacterial activity against *Escherichia coli* and *Bacillus subtilis* when compared to standard drug (Penicillin) [9]. Compound **B** displayed excellent antifungal activity against *Fusarium oxysporum* when compared with Amphotericin B [10]. Compound **C** exhibited remarkably high antimicrobial activities against *Klebsiella pneumoniae* and *Fusarium oxysporum* [11]. Furthermore, the pyrazolo[1,5-*a*]pyrimidine moiety is found in some marketed drugs with different biological activities such as: Zaleplon, which is a sedative-hypnotic used to treat insomnia; Ocinaplon, which is an anxiolytic drug; and Dinaciclib (SCH-727965), a cyclin-dependent kinases (CDKs) inhibitor that it is being evaluated in clinical trials for various cancer indications [12,13] (Figure 1).

**Figure 2:**
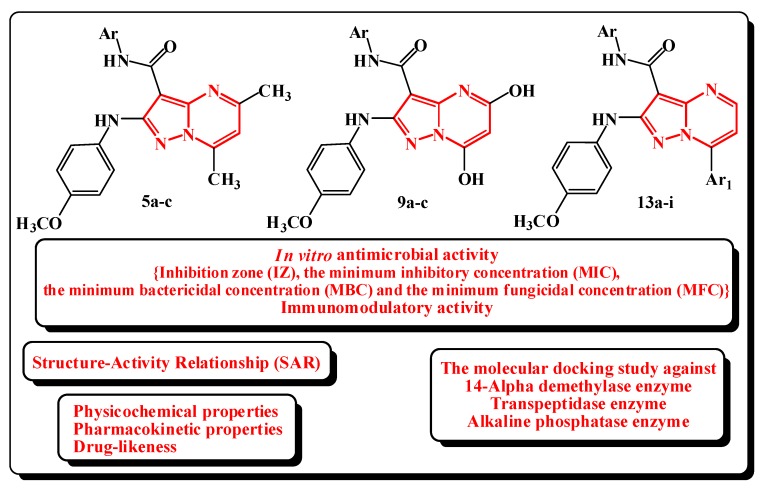
Structure of the target pyrazolo[1,5-*a*]pyrimidines (**5a**–**c**, **9a**–**c** and **13a**–**i**) and their studies.

Based on the above facts and in continuation of our target [14,15,16,17,18,19,20,21,22,23,24,25], the purpose of this paper is to evaluate and study the antimicrobial activity {inhibition zone (IZ), the minimum inhibitory concentration (MIC), the minimum bactericidal concentration (MBC) and the minimum fungicidal concentration (MFC)}, the immunomodulatory activity, the physicochemical, pharmacokinetic, and drug-likeness properties, the structure–activity relationship and the molecular docking of pyrazolo[1,5-*a*]pyrimidine derivatives (e.g., 5,7-dimethylpyrazolo[1,5-*a*]pyrimidines **5a**–**c**, 5,7-dihydroxypyrazolo[1,5-*a*]pyrimidines **9a**–**c** and 7-aryl-pyrazolo[1,5-*a*]pyrimidine **13a**–**i,** Figure 2.

## 2. Results and Discussion

### 2.1. Chemistry

**Scheme 1:**
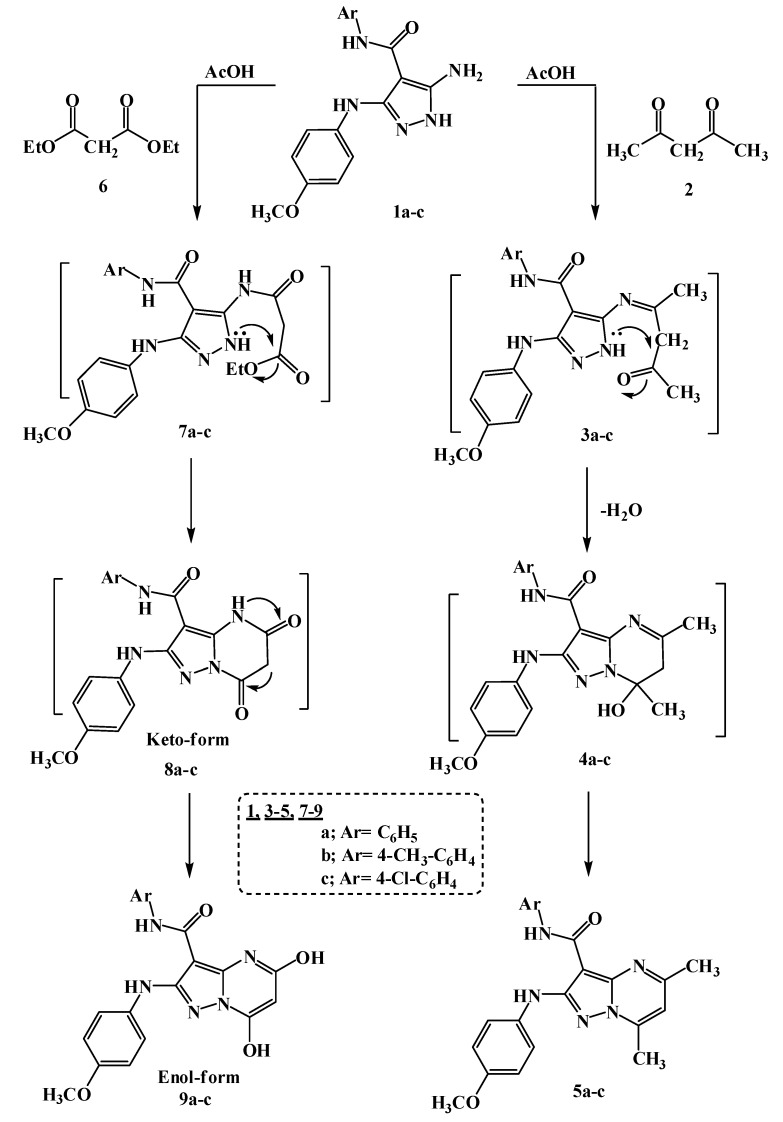
Synthesis of pyrazolo[1,5-*a*]pyrimidines **5a**–**c** and **9a**–**c**.

**Scheme 2:**
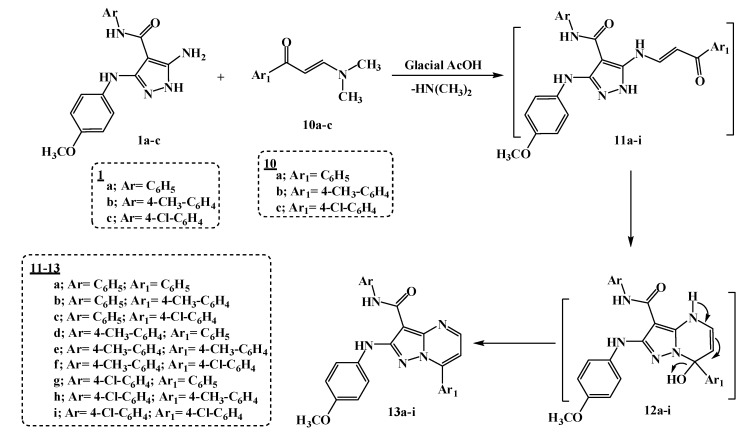
Synthesis of 7-aryl-pyrazolo[1,5-*a*]pyrimidines **13a**–**i**.

The starting materials, 5-amino-*N*-aryl-1*H*-pyrazole-4-carboxamides **1a**–**c**, were prepared according to our previous work [26]. The syntheses of the targets, 5,7-dimethylpyrazolo[1,5-*a*]pyrimidines **5a**–**c**, 5,7-dihydroxypyrazolo[1,5-*a*]pyrimidines **9a**–**c** and 7-aryl-pyrazolo[1,5-*a*]pyrimidines **13a**–**i**, are illustrated in Scheme 1 and Scheme 2.

Compounds **1a**–**c** were reacted with acetylacetone (2) or diethyl malonate (6) in refluxing glacial acetic acid to afford the corresponding 5,7-dimethylpyrazolo[1,5-*a*]pyrimidines **5a**–**c** or 5,7-dihydroxypyrazolo[1,5-*a*]pyrimidines **9a**–**c** (enol-form), respectively (Scheme 1).

The target compounds, 7-aryl-pyrazolo[1,5-*a*]pyrimidine **13a**–**i**, were prepared via the reaction of compounds **1a**–**c** with 1-(aryl)-3-(dimethylamino)prop-2-en-1-ones **10a**–**c** in glacial CH_3_COOH (Scheme 2).

### 2.2. Biological Evaluation

#### 2.2.1. In Vitro Antimicrobial Evaluation

The antimicrobial activities inhibition zone (IZ, mm ± standard deviation) of the pyrazolo[1,5-*a*]pyrimidines (**5a**–**c**, **9a**–**c** and **13a**–**i**) were evaluated using the agar plate diffusion method [27,28]. The results of the inhibition zone are listed in Table 1.

From the results (Table 1), we can deduce that eight compounds (**5c**, **9a**, **9c**, **13a**, **13c**, **13d**, **13e** and **13h**) displayed broad-spectrum in vitro antimicrobial activities against the bacteria and fungi used in this study. Therefore, the minimum inhibitory concentration (MIC, µg/mL), the minimum bactericidal concentration (MBC, µg/mL) and the minimum fungicidal concentration (MFC, µg/mL) of the most potent pyrazolo[1,5-*a*]pyrimidines (**5c**, **9a**, **9c**, **13a**, **13c**, **13d**, **13e** and **13h**) were determined by the conventional technique termed paper disk diffusion [29,30,31] and the results of MIC, MBC and MFC are listed in Table 2.

From the result of MIC values, we could see that in the case of *Bacillus cereus* (Bc) bacteria, pyrazolopyrimidine **9a** (MIC = 3.55 µg/mL) was 1-fold more potent than Amoxicillin/Clavulanic acid (MIC = 7.81 µg/mL), while compounds **9c** (MIC = 5.73 µg/mL), **13e** (MIC = 7.43 µg/mL) and **13h** (MIC = 7.47 µg/mL) were equipotent to the reference drug (Amoxicillin/Clavulanic acid (MIC = 7.81 µg/mL)).

In the case of *Staphylococcus aureus* (Sa) bacteria, the two compounds **5c** (MIC = 3.9 µg/mL) and **9c** (MIC = 2.95 µg/mL) were equipotent to Amoxicillin/Clavulanic acid (MIC = 3.12 µg/mL).

In the case of *Enterococcus faecalis* (Ef) bacteria, pyrazolopyrimidine **9c** (MIC = 1.85 µg/mL vs. MIC = 3.9 µg/mL) was 1-fold more potent than the drug used, while compound **5c** (MIC = 3.9 µg/mL) was equipotent to Amoxicillin/Clavulanic acid (MIC = 3.9 µg/mL).

The two compounds **13d** (MIC = 3.39 µg/mL) and **13h** (MIC = 3.71 µg/mL) were 1-fold more potent than the Amoxicillin/Clavulanic acid (MIC = 5.68 µg/mL) in the case of *Escherichia coli* (Ec) bacteria.

The five compounds **5c** (MIC = 3.9 µg/mL), **9c** (MIC = 6.5 µg/mL), **13d** (MIC = 6.6 µg/mL), **13e** (MIC = 7.14 µg/mL) and **13h** (MIC = 6.28 µg/mL) were more potent than the drug used (Amoxicillin/Clavulanic acid (MIC = 9.46 µg/mL)) in the case of *Pseudomonas aeruginosa* (Pa) bacteria.

For the *Salmonella typhi* (St) bacteria, the compounds **5c** (MIC = 3.39 µg/mL) and **9c** (MIC = 2.83 µg/mL) were 1-fold more potent than the standard drug Amoxicillin/Clavulanic acid (MIC = 6.79 µg/mL). The four compounds **9a** (MIC = 7.1 µg/mL), **13a** (MIC = 7.47 µg/mL), **13d** (MIC = 6.79 µg/mL) and **13h** (MIC = 6.28 µg/mL) were nearly equipotent to reference drug Amoxicillin/Clavulanic acid (MIC = 6.79 µg/mL).

The antifungal activity of the pyrazolo[1,5-*a*]pyrimidines on the *Candida albicans* (Ca) fungi showed that three of the pyrazolo[1,5-*a*]pyrimidines (**9c** (MIC = 3.39 µg/mL), **13e** (MIC = 3.39 µg/mL) and **13h** (MIC = 2.95 µg/mL)) were 1-fold more potent than the antifungal standard drug Nystatin (MIC = 7.81 µg/mL). Furthermore, the three compounds **5c**, **9a** and **13d** were equipotent to the reference drug used.

On estimation of the antifungal activity on the *Fusarium oxysporum* (Fo) fungi, the compounds **13d** (MIC = 20.83 µg/mL), **13e** (MIC = 24.79 µg/mL) and **13h** (MIC = 22.88 µg/mL) were more potent than the reference drug Nystatin (MIC = 26.4 µg/mL). The two compounds **9a** and **9c** were nearly equipotent to the reference drug Nystatin.

In the case of the *Aspergillus brasiliensis* (Ab) fungi, the standard drug Nystatin (MIC = 20.2 µg/mL) was more active than all the tested pyrazolo[1,5-*a*]pyrimidines (**5a**–**c**, **9a**–**c** and **13a**–**i**), with their MIC ranging from 21.25 µg/mL to 96.15 µg/mL.

In order to further study and conclude which of the above promising pyrazolopyrimidines compounds **5c**, **9a**, **9c**, **13a**, **13c**, **13d**, **13e** and **13h** may be bactericidal or show bacteriostatic action, the action of the compounds will be deduced from the relationship between MIC and MBC or MFC and from a comparison between the values of the compounds (Table 2). The range ratio between MIC-MBC was 1–2 ratios; antibacterial agents are generally regarded as bactericidal if the MBC is no more than four times the MIC [32,33], and the values of the compounds are in this range. Therefore, the results indicated that the pyrazolopyrimidine compounds exhibited bactericidal and fungicidal properties in comparison to Amoxicillin/Clavulanic acid as an antibacterial standard and Nystatin as an antifungal standard.

#### 2.2.2. Immunomodulatory Activity for Active Compounds

In this study, the immunomodulatory activity of the active pyrazolopyrimidine compounds was investigated by in vitro test. The most potent compounds depending on the previous antimicrobial results were chosen to evaluate their immunomodulatory activity for it was predicted that these compounds may have a dual function. The neutrophils play a primary role as an effecting or killer cell for many types of infections [34].

The active pyrazolopyrimidine compounds **5c**, **9a**, **9c**, **13a**, **13c**, **13d**, **13e** and **13h** were evaluated by nitroblue tetrazolium (NBT) reduction test, and the results were presented as intracellular killing percentage % values and listed in Table 3.

The highest immunostimulatory action were **13d**, **13e** and **9a** with 136.5 ± 0.3, 129.8 ± 0.47 and 125.6 ± 0.44, respectively. In various in vitro and in vivo bioassays, Zymosan represents an efficient chemo-attractant parameter, where the nucleophile can cause microorganism intracellular killing.

From Table 3, an increase in the intracellular killing activity of neutrophils can be observed. Therefore, the effectiveness of the body’s immune system may be activated by these compounds, as the neutrophils play a primary role as an effecting or killer cell for many types of infections.

### 2.3. Structure–Activity Relationship (SAR)

**Figure 3:**
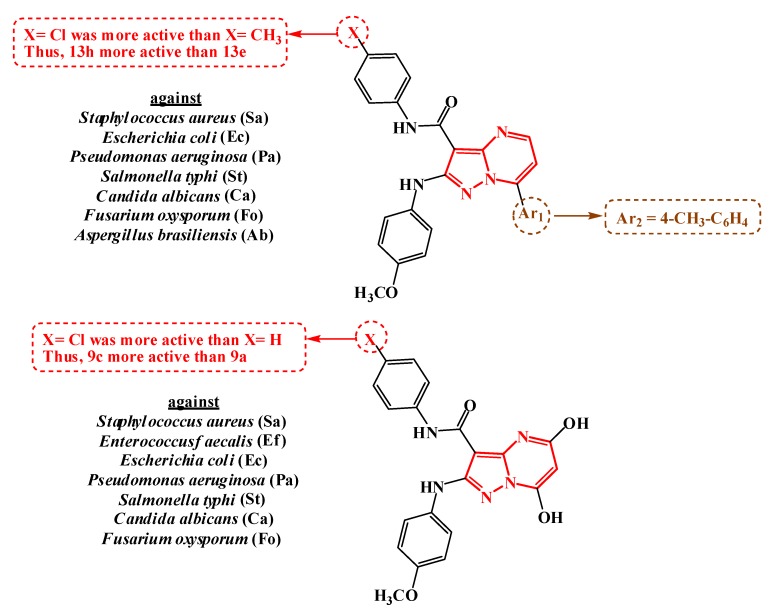
A brief structure–activity relationship (SAR) study of the active pyrazolopyrimidines.

From the results (Table 2) of in vitro antimicrobial activities of pyrazolopyrimidine compounds **5c**, **9a**, **9c**, **13a**, **13c**, **13d**, **13e** and **13h** against the screening organisms, it was found that some pyrazolopyrmidine derivatives bearing X = Cl (Chloro atom, electron withdrawing group) were more active than those bearing X = CH_3_ (Methyl group, electron donating group), whereas **13h** was more active than **13e**. Furthermore, **9c** was more active than **9a**, where the derivatives bearing X = Cl (Chloro atom) were more active than those bearing X = H (Hydrogen atom, without substitutions) against some of the screening organisms (Figure 3).

### 2.4. Physicochemical, Pharmacokinetic, ADME, Toxicity Prediction and Drug-Likeness

#### 2.4.1. Lipinski’s Rule of Five for Pyrazolo[1,5-*a*]pyrimidines **5a**–**c**, **9a**–**c** and **13a**–**i**

To qualify 5,7-dimethylpyrazolo[1,5-*a*]pyrimidines **5a**–**c**, 5,7-dihydroxypyrazolo[1,5-*a*]pyrimidines **9a**–**c** and 7-aryl-pyrazolo[1,5-*a*]pyrimidine **13a**–**i** as drug candidates, the computed molecular properties of Lipinski’s rule of five were calculated using SwissADME web (http://swissadme.ch/index.php#undefined) and are shown in Table 4.

The number of hydrogen bond acceptors and donors for all the pyrazolo[1,5-*a*]pyrimidines **5a**–**c**, **9a**–**c** and **13a**–**i** were in accordance with Lipinski’s rule of five. The molecular weight (MW) of the pyrazolo[1,5-*a*]pyrimidines **5a**–**c**, **9a**–**c** and **13a**–**i** were in range (less than 500, Lipinski’s rule), except **13i** which has MW = 504.37 g/mol. The lipophilicity property (MLogP ≤ 4.15, octanol-water partition coefficient) was in the range for all the pyrazolo[1,5-*a*]pyrimidines **5a**–**c**, **9a**–**c** and **13a-i** excluding **13c**, **13e**, **13f**, **13g**, **13h** and **13i**. The highly lipophilic character (MLogP > 4.15) in the range between 4.22–4.76 of the compounds **13c**, **13e**, **13f**, **13g**, **13h** and **13i** may be because of the presence of a chloro atom in their structures [35].

#### 2.4.2. Pharmacokinetic Properties of Pyrazolo[1,5-*a*]pyrimidines **5a–c**, **9a–c** and **13a–i**

The computed pharmacokinetic properties of the series of pyrazolo[1,5-*a*]pyrimidines **5a–c**, **9a**–**c** and **13a**–**i** were calculated using SwissADME web (http://swissadme.ch/index.php#undefined). The results are shown in Table S1 (see Supplementary Material).

All the compounds **5a**–**c**, **9a**–**c** and **13a**–**i** show high gastrointestinal absorption (GI absorption) except **13i**. All the compounds **5a**–**c**, **9a**–**c** and **13a**–**i** are not predicted to penetrate the blood–brain barrier (BBB) and are non-substrates for P-glycoprotein (P-gp). Therefore, they have no effect on the central nervous system.

Inhibition of the five major CYP isoforms (CYP1A2, CYP2C19, CYP2C9, CYP2D6 and CYP3A4) is certainly one major cause of pharmacokinetic-related drug–drug interactions. The compounds **13b–i** are non-inhibitors of the CYP1A2 enzyme, the two compounds **9a** and **9b** are non-inhibitors of the CYP2C19 enzyme and all compounds **5a**–**c**, **9a**–**c** and **13a**–**i** are inhibitors and are active against the CYP2C9 enzyme. The series **9a**–**c** are non-inhibitors of the CYP2D6 enzyme and compound **9a** is a non-inhibitor of the CYP3A4 enzyme [36].

#### 2.4.3. In Silico ADME and Toxicity Prediction of Pyrazolo[1,5-*a*]pyrimidines **5a**–**c**, **9a**–**c** and **13a**–**i**

The computed in silico ADME and toxicity predictions of pyrazolo[1,5-*a*]pyrimidines **5a**–**c**, **9a**–**c** and **13a**–**i** were calculated using the PreADMET web (https://preadmet.bmdrc.kr). The results are shown in Tables S2 and S3 (see Supplementary Material).

On the human intestinal absorption (HIA) test, all the pyrazolo[1,5-*a*]pyrimidines **5a**–**c**, **9a**–**c** and **13a**–**i** expressed more than 70% human intestinal absorption (HIA) values, indicating good permeation across the membrane.

The results of the in vitro Caco-2 cell permeability indicated that all pyrazolo[1,5-*a*]pyrimidines **5a**–**c**, **9a**–**c** and **13a**–**i** exhibited moderate permeation.

On the in vitro MDCK cell permeability test, all the pyrazolo[1,5-*a*]pyrimidines **5a**–**c**, **9a**–**c** and **13a**–**i** showed permeation less than 25 nm/s, indicating low permeability.

For the in vitro skin permeability test for the delivery of drugs via transdermal administration, all the pyrazolo[1,5-*a*]pyrimidines **5a**–**c**, **9a**–**c** and **13a**–**i** exhibited negative values.

For the in vitro plasma protein binding (PPB) test, most of the pyrazolo[1,5-*a*]pyrimidines were predicted more than 90%, which indicates decreased excretion and increased half-life.

On the Ames test that assesses mutagenicity, all the pyrazolo[1,5-*a*]pyrimidines were predicted to be mutagens. Moreover, on analyzing carcinogenicity in animals (mouse), all the compounds were predicted as positive, except compound **5b** which presented negative, while for the carcinogenicity test in animals (rat), the compounds **5c**, **9a**–**c**, **13c** and **13f**–**i** were predicted as negative.

In the case of the hERG encodes potassium channels test, compound **9a** presented high risk; **13a** presented ambiguous risk and the rest of the compounds presented medium risk.

#### 2.4.4. Drug Likeness Calculations of Pyrazolo[1,5-*a*]pyrimidines **5a**–**c**, **9a**–**c** and **13a**–**i**

Molecular polar surface area (TPSA (A^2^)) is an affected parameter in the prediction of drug transport properties. Molecular volume was calculated by using the MolSoft website (http://molsoft.com/mprop/). The percentage of absorption (%ABS) was calculated by using %ABS = 109 − (0.345 × TPSA) and is referred to the degree of absorption [37]. The series of pyrazolo[1,5-*a*]pyrimidines **5a**–**c**, **9a**–**c** and **13a**–**i** possessed the percentage of absorption (%ABS) of 88.24%, 76.53% and 88.45%, respectively.

Computed drug-likeness scores of the compounds pyrazolo[1,5-*a*]pyrimidines, **5a**–**c**, **9a**–**c** and **13a**–**i** are presented in Table 5. Compound **5b** has less value (DLS = 0.40) but the two compounds **13c** and **13i** possessed a maximum drug-likeness model score of 1.31 and 1.44, respectively.

### 2.5. Molecular Docking

#### 2.5.1. Bacteria

**Figure 4:**
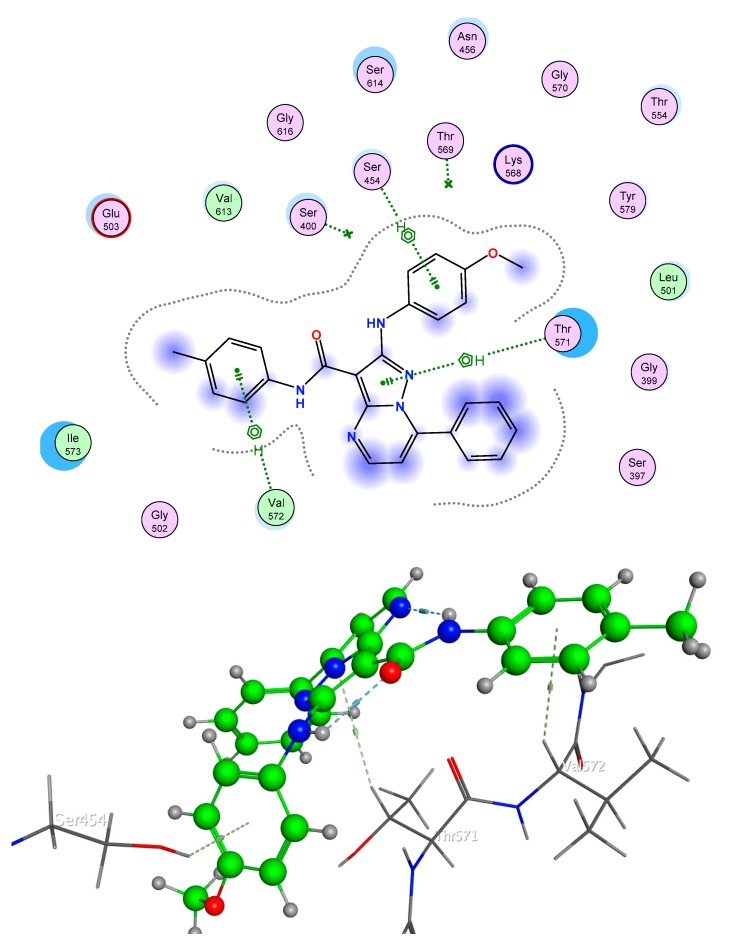
2D and 3D interaction diagrams of compound **13d** with transpeptidase.

There are about 30 enzymes involved in the biochemistry of the cell wall of bacteria. Antibiotics such as the penicillin series work on the inhibition of the final cross link step by inhibiting the transpeptidase enzyme. The E-score (energy score), considered as one of the most important factors, reflects the interaction between the ligand and enzyme. The molecular docking validation explains the interaction (E-score) between the reference ligand, 2-[*N*-cyclohexylamino]ethane sulfonic acid, (E-score = −5.23) and the compounds (**5c**, **9a**, **9c**, **13a**, **13c**, **13d**, **13e** and **13h**) with a transpeptidase enzyme as −6.56, −6.47, −6.82, −6.44, −6.50, −7.17, −6.97 and −6.87, respectively. Furthermore, Figure 4 shows the 2D and 3D interaction diagrams of compound **13d** with transpeptidase.

#### 2.5.2. Fungi

**Figure 5:**
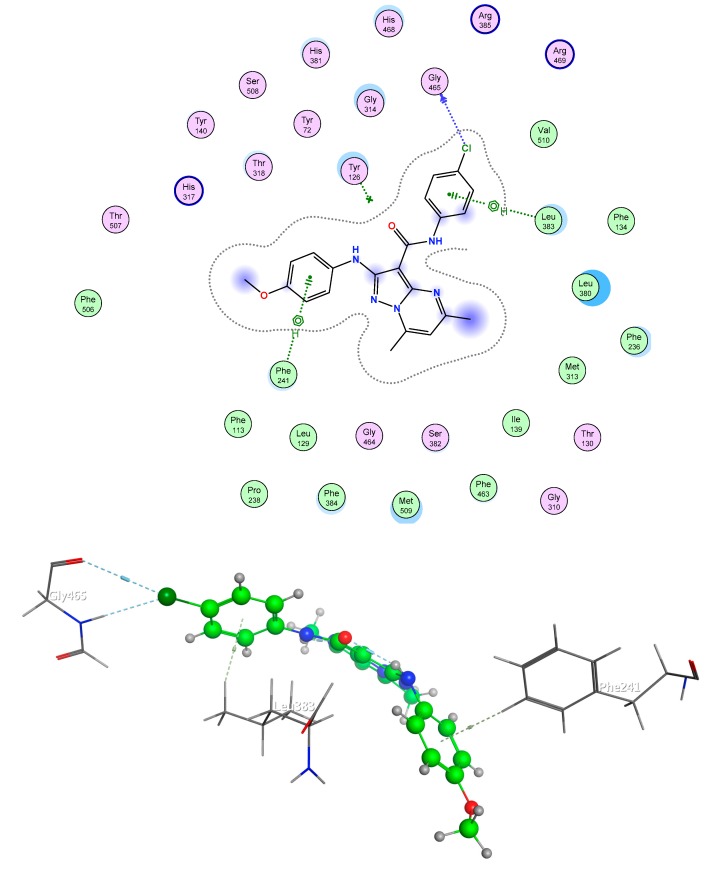
2D and 3D interaction diagrams of compound **5c** with 14-α demethylase.

Ergosterol biosynthesis is considered as a very important step in the building of the fungal cell membrane. 14-Alfa demethylase is the responsible enzyme that converts lanosterol to ergosterol. The molecular docking study (E-score) between the reference ligand, lanosterol, (E-score = −8.06) and the synthesized compounds (**5c**, **9a**, **9c**, **13a**, **13c**, **13d**, **13e** and **13h**) with 14-alpha demethylase showed figures of −8.15, −7.40, −7.95, −7.8, −8.12, −8.54, −8.24 and −8.17, respectively. Figure 5 shows 2D and 3D interaction diagrams of compound **5c** with 14-α demethylase.

#### 2.5.3. Immunity Docking

Nitro blue tetrazolium (NBT) is an organic salt compound purchased as the chloride salt based on two tetrazole moieties. These moieties have sensitivity to alkaline phosphatase (ALP) enzyme and therefore are used as a test in immunology to detect the reactivity of organic compounds on the immunity system. ALP enzyme is an ahomodimeric metalloenzyme promoting the unspecified hydrolysis of the phosphate monoesters process. This enzymatic promotion proceeds by a phosphoseryl intermediate to give inorganic phosphate and an alcohol. ALP enzyme structure as a phosphate has been determined by X-ray technique [38]. The inhibition of ALP presents a unique challenge since the active site pocket is characteristically shallow and, in continuation of our work [39,40,41] to discover new drug enzyme interactions, we present this calculated part.

**Figure 6:**
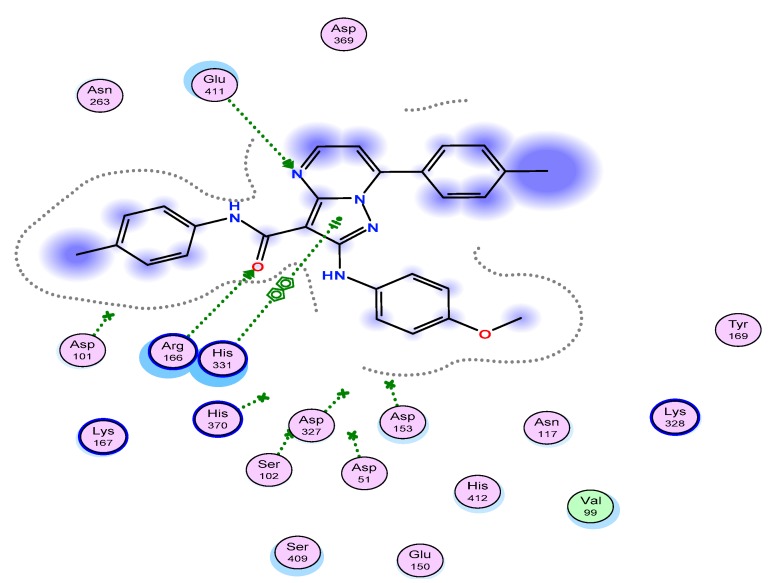
2D interaction diagrams of compound **13e** with alkaline phosphatase (ALP).

The active site analysis of the ALP protein was performed from a database of similar amino acid residues (Glu411, Arg166, His331, Asp269, Lys328, Asn263, Asp153, Asp327, His412, Ser102, Gly150, Asp51, His370 and Tyr169). The 2D pocket in the alkaline phosphatase (ALP) complex with **13e** as a ligand explained that the interaction between them is potent through hydrogen bonds. Furthermore, Figure 6 presents the 2D the interaction between **13e** as a ligand with alkaline phosphatase (ALP) residues.

#### 2.5.4. Pharmacophore and Electrostatic Map of **13e**

**Figure 7:**
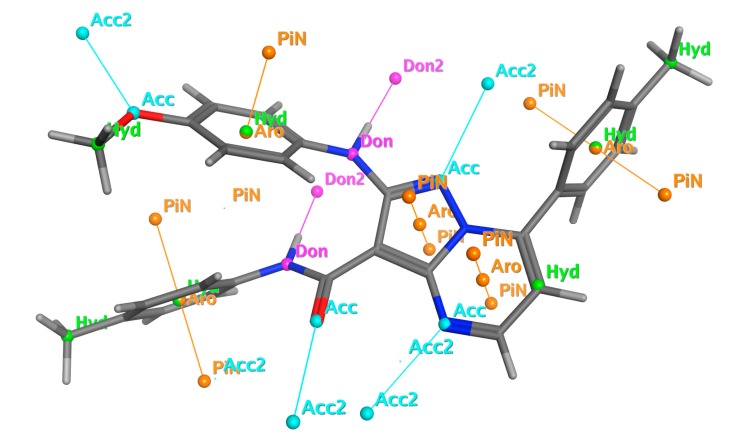
3D pharmacophore structure of **13e** simulated to an active site in alkaline phosphatase (ALP).

**Figure 8:**
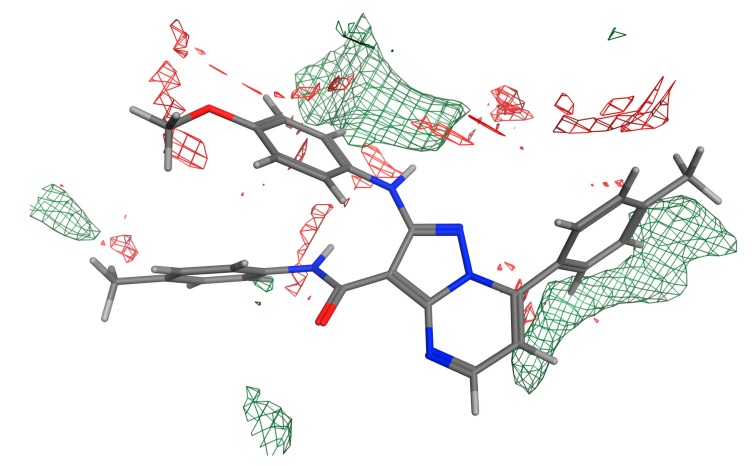
The electrostatic map of **13e** shows the hydrophilic part (violet color) and the lipophilic part (blue color).

The selection of pyrazolopyrimidine scaffold analogs (**5a**–**c**, **9a**–**c**, **13a**–**i**) to build a pharmacophore for potential alkaline phosphatase (ALP) inhibitors was based on the high potency superposition of **13e** that generated a pharmacophore with H-bond acceptors (Acc, Acc2), with H-bond donor projection (Don), an aromatic center (Aro) and which was hydrophobic (hyd) (Figure 7). The electrostatic map of compound **13e** shows the hydrophilic section as a violet color and the lipophilic part as a blue color (Figure 8).

## 3. Materials and Methods

### 3.1. Chemicals

*N*-Aryl-2-[(4-methoxyphenyl)amino]-5,7-dimethylpyrazolo[1,5-*a*]-pyrimidine-3-carboxamides **5a**–**c** [42], *N*-aryl-5,7-dihydroxy-2-(4-methoxyphenylamino)pyrazolo[1,5-*a*]pyrimidine-3-carboxamides **9a**–**c** [43] and 7-aryl-2-(arylamino)pyrazolo[1,5-*a*]pyrimidine-3-carboxamides **13a**–**i** [44] were prepared according to the reported procedure.

The chemical structures of **5a**–**c** [42], **9a**–**c** [43] and **13a**–**i** [44] were confirmed via spectral data (see Supplementary Material).

### 3.2. In Vitro Biological Evaluation

The test microorganisms used in this study were: Gram-positive bacteria: *Bacillus cereus* (ATCC14579, Bc), *Staphylococcus aureus* (ATCC 29213, Sa) and *Enterococcus faecalis* (ATCC 29212, Ef). Gram-negative bacteria: *Escherichia coli* (ATCC 25922, Ec), *Pseudomonas aeruginosa* (ATCC 27853, Pa) and *Salmonella typhi* (ATCC 6539, St). Fungi: *Candida albicans* (ATCC 10231, Ca), *Fusarium oxysporum* (RCMB 008002, Fo) and *Aspergillus brasiliensis* (ATCC 16404, Ab).

The antimicrobial activities inhibition zone (IZ, mm ± standard deviation) was measured according to the agar plate diffusion method [27,28] (see Supplementary Material).

The MIC, MBC and MFC of the potent pyrazolo[1,5-*a*]pyrimidines: The minimum inhibitory concentration (MIC), the minimum bactericidal concentration (MBC, µg/mL) and the minimum fungicidal concentration (MFC, µg/mL) of the most potent pyrazolo[1,5-*a*]pyrimidines (**5c**, **9a**, **9c**, **13a**, **13c**, **13d**, **13e** and **13h**) were determined by the conventional technique termed paper disk diffusion [29,30,31] (see Supplementary Material).

The immunomodulatory activity of the potent pyrazolo[1,5-*a*]pyrimidines compounds **5c**, **9a**, **9c**, **13a**, **13c**, **13d**, **13e** and **13h** was evaluated by nitroblue tetrazolium (NBT) reduction test [45,46] (see Supplementary Material).

### 3.3. Molecular Docking

The standard docking protocol was carried out using MOE 2015.10 software. The proteins in the mdb file downloaded from the PDB (protein data bank) were transpeptidase (PDB code: 4ZTK), 14-alpha demethylase (PDB code: 4LXJ) and alkaline phosphatase ALP (PDB code: 1EW8) (http://www.rcsb.org/pdb/home/home.do).

## 4. Conclusions

In this work, a series of pyrazolo[1,5-*a*]pyrimidine derivatives **5a**–**c**, **9a**–**c** and **13a**–**i** were synthesized for evaluation of their in vitro antimicrobial and immunomodulatory activities. The result of pyrazolo[1,5-*a*]pyrimidines exhibited that most of the compounds displayed significant antimicrobial (bactericidal and fungicidal properties) and immunomodulatory activities. Furthermore, the in silico predicted physicochemical, pharmacokinetic, ADMET properties and drug-likeness studies of the pyrazolo[1,5-*a*]pyrimidines **5a**–**c**, **9a**–**c** and **13a**–**i** revealed that the compounds fulfill Lipinski’s rule requirements and have good drug score values, particularly in **13c** (DLS = 1.31) and **13i** (DLS = 1.44). Furthermore, the molecular docking study was compatible with antimicrobial and immunomodulatory activities. These preliminary results of pyrazolo[1,5-*a*]pyrimidines as antimicrobial activities and the structure–activity relationship with molecular docking could provide an exceptional model that may lead to the discovery of new drugs.

## Figures and Tables

**Table 1:** Antimicrobial activities of the pyrazolo[1,5-*a*]pyrimidines (**5a**–**c**, **9a**–**c** and **13a**–**i**) and the reference drugs expressed as inhibition zone (IZ, mm ± standard deviation).

Comp.	*Gram-Positive Bacteria*			*Gram-Negative Bacteria*			*Fungi*		
	Bc	Sa	Ef	Ec	Pa	St	Ca	Fo	Ab
**5a**	11 ± 0.61	20 ± 0.25	13 ± 0.23	13 ± 0.34	NA	NA	NA	NA	NA
**5b**	14 ± 0.54	11 ± 0.84	13 ± 0.23	10 ± 0.62	NA	NA	12 ± 0.63	7 ± 0.34	NA
**5c**	19 ± 0.14	21 ± 0.49	22 ± 0.68	18 ± 0.24	19 ± 0.43	18 ± 0.39	17 ± 0.33	12 ± 0.95	13 ± 0.95
**9a**	23 ± 0.18	19 ± 0.37	16 ± 0.83	14 ± 0.21	16 ± 0.33	16 ± 0.77	15 ± 0.25	13 ± 0.39	16 ± 0.79
**9b**	10 ± 0.24	15 ± 0.15	NA	18 ± 0.18	NA	14 ± 0.48	NA	10 ± 0.28	NA
**9c**	16 ± 0.78	21 ± 0.24	25 ± 0.23	17 ± 0.84	15 ± 0.34	18 ± 0.18	19 ± 0.24	16 ± 0.78	13 ± 0.43
**13a**	13 ± 0.11	16 ± 0.34	13 ± 0.23	15 ± 0.23	14 ± 0.78	16 ± 0.33	13 ± 0.65	14 ± 0.11	11 ± 0.42
**13b**	9 ± 0.37	NA	NA	12 ± 0.62	NA	NA	12 ± 0.46	14 ± 0.22	NA
**13c**	17 ± 0.23	10 ± 0.47	13 ± 0.23	15 ± 0.45	12 ± 0.61	14 ± 0.47	14 ± 0.23	11 ± 0.22	10 ± 0.32
**13d**	14 ± 0.23	18 ± 0.87	16 ± 0.23	19 ± 0.14	17 ± 0.46	17 ± 0.76	16 ± 0.81	15 ± 0.66	13 ± 0.33
**13e**	17 ± 0.32	17 ± 0.74	23 ± 0.21	16 ± 0.96	15 ± 0.77	13 ± 0.98	18 ± 0.11	13 ± 0.44	12 ± 0.32
**13f**	10 ± 0.14	15 ± 0.64	NA	11 ± 0.12	NA	13 ± 0.32	12 ± 0.44	10 ± 0.48	NA
**13g**	12 ± 0.47	8 ± 0.2	13 ± 0.23	11 ± 0.27	NA	NA	NA	NA	NA
**13h**	20 ± 0.34	18 ± 0.16	20 ± 0.13	17 ± 0.15	18 ± 0.12	16 ± 0.85	20 ± 0.26	15 ± 0.76	14 ± 0.12
**13i**	NA	NA	NA	13 ± 0.85	NA	11 ± 0.12	15 ± 0.56	12 ± 0.15	NA
**ST1**	22 ± 0.42	24 ± 0.61	25 ± 0.45	23 ± 0.33	20 ± 0.55	22 ± 0.18	NA	NA	NA
**ST2**	NA	NA	NA	NA	NA	NA	15 ± 0.2	20 ± 0.32	18 ± 0.2

Gram-positive bacteria: *Bacillus cereus* (ATCC14579, Bc), *Staphylococcus aureus* (ATCC 29213, Sa) and *Enterococcus faecalis* (ATCC 29212, Ef). Gram-negative bacteria: *Escherichia coli* (ATCC 25922, Ec) *Pseudomonas aeruginosa* (ATCC 27853, Pa) and *Salmonella typhi* (ATCC 6539, St). Fungi: *Candida albicans* (ATCC 10231, Ca), *Fusarium oxysporum* (RCMB 008002, Fo) and *Aspergillus brasiliensis* (ATCC 16404, Ab). ST1: Amoxicillin/Clavulanic acid as antibacterial standard; ST2: Nystatin as antifungal standard. NA: No activity.

**Table 2:** The minimum inhibitory concentration (MIC, µg/mL), the minimum bactericidal concentration (MBC, µg/mL) and the minimum fungicidal concentration (MFC, µg/mL) of the pyrazolo[1,5-*a*]pyrimidines (**5a**–**c**, **9a**–**c** and **13a**–**i**) and the reference drugs.

Comp.	*Gram-Positive Bacteria*						*Gram-Negative Bacteria*						*Fungi*					
	*Bc*		*Sa*		*Ef*		*Ec*		*Pa*		*St*		*Ca*		*Fo*		*Ab*	
	MIC	MBC	MIC	MBC	MIC	MBC	MIC	MBC	MIC	MBC	MIC	MBC	MIC	MFC	MIC	MFC	MIC	MFC
**5c**	31.25	62.5	3.9	7.81	3.9	7.81	6.79	15.62	3.9	7.81	3.39	7.81	7.14	16.44	52.08	62.5	29.46	41.25
**9a**	3.55	7.81	6.28	13.2	7.1	15.62	7.14	16.44	14.16	29.75	7.1	15.62	7.43	15.62	27.9	44.65	21.25	29.76
**9c**	5.73	13.2	2.95	6.51	1.85	3.9	6.6	13.2	6.5	15.62	2.83	6.51	3.39	7.81	27.77	41.66	24.03	31.25
**13a**	18.93	41.66	29.76	62.5	19.83	41.66	7.47	16.44	12.93	29.75	7.47	16.44	15.62	31.25	32.04	41.66	41.66	62.5
**13c**	29.76	62.5	56.81	125	31.25	62.5	6.79	15.62	13.02	31.25	13.52	29.75	11.27	24.8	52.08	62.5	96.15	125
**13d**	14.87	29.75	14.2	31.25	14.2	31.25	3.39	7.81	6.6	13.2	6.79	15.62	7.1	15.62	20.83	31.25	26.04	41.66
**13e**	7.43	15.62	18.09	41.66	12.93	29.75	14.87	29.75	7.14	16.44	15.62	31.25	3.39	7.81	24.79	34.71	31.89	44.65
**13h**	7.47	16.44	7.1	15.62	13.58	31.25	3.71	7.81	6.28	13.2	6.28	13.2	2.95	6.51	22.88	29.75	24.03	31.25
**ST1**	7.81	15.62	3.12	6.24	3.9	7.81	5.68	12.5	9.46	20.83	6.79	15.62	-	-	-	-	-	-
**ST2**	-	-	-	-	-	-	-	-	-	-	-	-	7.81	15.62	26.4	31.25	20.2	24.13

Gram-positive bacteria: *Bacillus cereus* (ATCC14579, Bc), *Staphylococcus aureus* (ATCC 29213, Sa) and *Enterococcus faecalis* (ATCC 29212, Ef). Gram-negative bacteria: *Escherichia coli* (ATCC 25922, Ec), *Pseudomonas aeruginosa* (ATCC 27853, Pa) and *Salmonella typhi* (ATCC 6539, St). Fungi: *Candida albicans* (ATCC 10231, Ca), *Fusarium oxysporum* (RCMB 008002, Fo) and *Aspergillus brasiliensis* (ATCC 16404, Ab). ST1: Amoxicillin/Clavulanic acid as antibacterial standard; ST2: Nystatin as antifungal standard.

**Table 3:** The intracellular killing activities of active pyrazolopyrimidine compounds.

Compounds	Intracellular Killing Activity %
**5c**	76.2 ± 0.23
**9a**	125.6 ± 0.44
**9c**	122.9 ± 0.79
**13a**	98.7 ± 0.61
**13c**	87.5 ± 0.33
**13d**	136.3 ± 0.16
**13e**	129.8 ± 0.47
**13h**	117.4 ± 0.98

**Table 4:** Lipinski’s rule of five for the compounds pyrazolo[1,5-*a*]pyrimidines **5a**–**c**, **9a**–**c** and **13a**–**i**.

Compounds	MW ^a^	*M*LogP ^b^	*n*HBA ^c^	*n*HBD ^d^	n*_violations_* ^f^
Rule	<500	≤4.15	≤10	≤5	0
**5a**	387.43	3.19	4	2	0
**5b**	401.46	3.41	4	2	0
**5c**	421.88	3.67	4	2	0
**9a**	391.38	2.13	6	4	0
**9b**	405.41	2.35	6	4	0
**9c**	425.83	2.62	6	4	0
**13a**	435.48	3.82	4	2	0
**13b**	449.50	4.02	4	2	0
**13c**	469.92	4.29	4	2	1
**13d**	449.50	4.02	4	2	0
**13e**	463.53	4.22	4	2	1
**13f**	483.95	4.49	4	2	1
**13g**	469.92	4.29	4	2	1
**13h**	483.95	4.49	4	2	1
**13i**	504.37	4.76	4	2	2

^a^ Molecular Weight; ^b^ Calculated Lipophilicity (MLog *P*_o/w_); ^c^ Number of Hydrogen Bond Acceptors;.^d^ Number of Hydrogen Bond Donors; ^e^ Violations from Lipinski’s Rule.

**Table 5:** Drug likeness calculations of the compounds pyrazolo[1,5-*a*]pyrimidines **5a**–**c, 9a**–**c** and **13a**–**i.**

Compounds	TPSA ^a^ (A^2^)	Volume ^b^ (A^3^)	%ABS ^c^ = 109 − (0.345 × TPSA)	Drug-Likeness Model Score (DLS)
**5a**	60.18	379.18	88.24	0.44
**5b**	60.18	400.12	88.24	0.40
**5c**	60.18	396.37	88.24	1.00
**9a**	94.13	358.28	76.53	0.61
**9b**	94.13	379.22	76.53	0.59
**9c**	94.13	375.47	76.53	1.15
**13a**	59.56	410.76	88.45	0.77
**13b**	59.56	431.70	88.45	0.73
**13c**	59.56	427.95	88.45	1.31
**13d**	59.56	431.70	88.45	0.62
**13e**	59.56	452.64	88.45	0.88
**13f**	59.56	448.89	88.45	1.25
**13g**	59.56	427.95	88.45	1.20
**13h**	59.56	448.89	88.45	1.25
**13i**	59.56	445.14	88.45	1.44

^a^ Topological polar surface area; ^b^ Molecular volume; ^c^ Percentage absorption.

## Supplementary Materials

The following are available online, Tables S1–S3, Spectral data of compounds (**1a**–**c**, **5a**–**c**, **9a**–**c** and **13a**–**i)** and biological methods are available online.
