# Supplementary material for: Biological Evaluation and Molecular Docking with In Silico Physicochemical, Pharmacokinetic and Toxicity Prediction of Pyrazolo[1,5-a]pyrimidines
Source: Molecules. 2020 Mar 21;25(6):1431. doi: 10.3390/molecules25061431 (PMC7144935; doi:10.3390/molecules25061431)
Supplement: Supplementary file 1 [file molecules-25-01431-s001.pdf]

SUPPLEMENTARY MATERIAL

For

Biological Evaluation and Molecular Docking with *In Silico* Physicochemical, Pharmacokinetic and Toxicity Prediction of Pyrazolo[1,5-*a*]pyrimidines

Ahmed M. Naglah[1,2], Ahmed A. Askar[3,*], Ashraf S. Hassan[4,*], Tamer K. Khatab[4], Mohamed A. Al-Omar[1] and Mashooq A. Bhat[5]

[1] Department of Pharmaceutical Chemistry, Drug Exploration and Development Chair (DEDC), College of Pharmacy, King Saud University, Riyadh 11451, Saudi Arabia; anaglah@ksu.edu.sa (A.M.N.); malomar1@ksu.edu.sa (M.A.A.-O.)

[2] Peptide Chemistry Department, National Research Centre, 12622-Dokki, Cairo, Egypt

[3] Botany and Microbiology Department, Faculty of Science (Boys), Al-Azhar University, Cairo 11751, Egypt; drahmed_askar@azhar.edu.eg

[4] Organometallic and Organometalloid Chemistry Department, National Research Centre, Dokki 12622 Cairo, Egypt; ashraf_salmoon@yahoo.com (A.S.H.); tamer_khatab@hotmail.com (T.K.K.)

[5] Department of Pharmaceutical Chemistry, College of Pharmacy, King Saud University, Riyadh 11451, Saudi Arabia; mabhat@ksu.edu.sa (M.A.B.)

*Correspondence: ashraf_salmoon@yahoo.com (A.S.H.); drahmed_askar@azhar.edu.eg (A.A.A.); Tel.: +20-100-664-5444 (A.S.H.); +20-101-081-5102 (A.A.A.)

Table S1: Pharmacokinetic properties of pyrazolo[1,5-*a*]pyrimidines **5a-c**, **9a-c** and **13a-i**

Compounds	GI absorption	BBB permeability	P-gp substrate	CYP isoenzymes				
				CYP1A2 inhibitor	CYP2C19 inhibitor	CYP2C9 inhibitor	CYP2D6 inhibitor	CYP3A4 inhibitor
**5a**	High	No	No	inhibitor	inhibitor	inhibitor	inhibitor	inhibitor
**5b**	High	No	No	inhibitor	inhibitor	inhibitor	inhibitor	inhibitor
**5c**	High	No	No	inhibitor	inhibitor	inhibitor	inhibitor	inhibitor
**9a**	High	No	No	inhibitor	non-inhibitor	inhibitor	non-inhibitor	non-inhibitor
**9b**	High	No	No	inhibitor	non-inhibitor	inhibitor	non-inhibitor	inhibitor
**9c**	High	No	No	inhibitor	inhibitor	inhibitor	non-inhibitor	inhibitor
**13a**	High	No	No	inhibitor	inhibitor	inhibitor	inhibitor	inhibitor
**13b**	High	No	No	non-inhibitor	inhibitor	inhibitor	inhibitor	inhibitor
**13c**	High	No	No	non-inhibitor	inhibitor	inhibitor	inhibitor	inhibitor
**13d**	High	No	No	non-inhibitor	inhibitor	inhibitor	inhibitor	inhibitor
**13e**	High	No	No	non-inhibitor	inhibitor	inhibitor	inhibitor	inhibitor
**13f**	High	No	No	non-inhibitor	inhibitor	inhibitor	inhibitor	inhibitor
**13g**	High	No	No	non-inhibitor	inhibitor	inhibitor	inhibitor	inhibitor
**13h**	High	No	No	non-inhibitor	inhibitor	inhibitor	inhibitor	inhibitor
**13i**	Low	No	No	non-inhibitor	inhibitor	inhibitor	inhibitor	inhibitor

GI, gastrointestinal; BBB, blood–brain barrier; P-gp, P-glycoprotein

Table S2: *In silico* ADME prediction of pyrazolo[1,5-*a*]pyrimidines **5a-c**, **9a-c** and **13a-i**

Compounds	Human intestinal absorption (%)	In vitro Caco-2 cell permeability (nm/s)	In vitro MDCK cell permeability (nm/s)	In vitro skin permeability (log K_p, cm/h)	In vitro plasma protein binding (%)
**Rule**	**0–20 (poor) 20–70 (moderate) 70–100 (well) <500**	**<4 (low) 4–70 (moderate) >70 (high)**	**<25 (low) 25–500 (moderate) >500 (high)**		**>90 (strongly bound) <90 (weakly bound)**
**5a**	95.13	38.92	0.21	-3.34	93.73
**5b**	95.29	40.68	0.05	-3.32	92.76
**5c**	95.81	26.84	0.27	-3.40	89.08
**9a**	88.19	17.76	0.05	-4.28	91.38
**9b**	88.67	18.53	0.05	-4.17	90.05
**9c**	90.39	20.24	0.23	-4.29	86.95
**13a**	96.16	39.15	0.10	-2.57	95.50
**13b**	96.27	40.61	0.08	-2.53	95.28
**13c**	96.59	29.75	0.06	-2.60	91.93
**13d**	96.27	40.77	0.05	-2.53	94.94
**13e**	96.36	42.05	0.05	-2.51	94.57
**13f**	96.66	30.86	0.05	-2.57	89.52
**13g**	96.59	29.67	0.06	-2.60	91.43
**13h**	96.66	30.79	0.05	-2.57	89.53
**13i**	96.86	31.92	0.05	-2.54	96.90

Table S3: *In silico* toxicity prediction of pyrazolo[1,5-*a*]pyrimidines **5a-c**, **9a-c** and **13a-i**

**Compounds**	**Ames test**	**Carcinogenicity (Mouse)**	**Carcinogenicity (rat)**	**hERG inhibition**
**5a**	Mutagen	Positive	Positive	Medium risk
**5b**	Mutagen	Negative	Positive	Medium risk
**5c**	Mutagen	Positive	Negative	Medium risk
**9a**	Mutagen	Positive	Negative	High risk
**9b**	Mutagen	Positive	Negative	Medium risk
**9c**	Mutagen	Positive	Negative	Medium risk
**13a**	Mutagen	Positive	Positive	Ambiguous
**13b**	Mutagen	Positive	Positive	Medium risk
**13c**	Mutagen	Positive	Negative	Medium risk
**13d**	Mutagen	Positive	Positive	Medium risk
**13e**	Mutagen	Positive	Positive	Medium risk
**13f**	Mutagen	Positive	Negative	Medium risk
**13g**	Mutagen	Positive	Negative	Medium risk
**13h**	Mutagen	Positive	Negative	Medium risk
**13i**	Mutagen	Positive	Negative	Medium risk

5-Amino-*N*-aryl-1*H*-pyrazole-4-carboxamides 1a-c:

5-Amino-3-(4-methoxyphenylamino)-N-phenyl-1H-pyrazole-4-carboxamide **(1a):** White crystals, m.p. 175-177 °C, yield (79%). IR (KBr) ν_{max}/cm^{-1} 3354, 3030 (NH, NH_2), 1645 (C=O), 1618 (C=N). ^1H-NMR (DMSO-d_6, δ ppm) 3.63 (s, 3H, OCH_3), 5.98 (s, 2H, NH_2, D_2O exchangeable), 6.76 (d, 2H, aromatic, *J*=7.7 Hz), 6.98 (t, 1H, aromatic, *J*=7.7 Hz), 7.18 (t, 2H, aromatic, *J*=8.4 Hz), 7.25 (d, 2H, aromatic, *J*=7.7 Hz), 7.45 (d, 2H, aromatic, *J*=7.7 Hz), 8.30 (s, 1H, NH, D_2O exchangeable), 8.74 (s, 1H, NH, D_2O exchangeable), 11.22 (s, 1H, NH, D_2O exchangeable). ^{13}C NMR (DMSO-d_6, δ ppm) 55.6 (-OCH_3), 88.3 (C_4, pyrazole), 114.7, 117.5, 120.3, 123.3, 129.1, 137.6, 139.4 (11C, aromatic), 149.1 (C_5, pyrazole), 150.9 (C_3, pyrazole), 153.1 (C, aromatic), 163.6 (C=O, amide). Anal. Calcd. (%) for $C_{17}H_{17}N_5O_2$ (323.35): C, 63.15; H, 5.30; N, 21.66. Found: C, 63.35; H, 5.15; N, 21.50 %.

5-Amino-3-(4-methoxyphenylamino)-N-(4-methylphenyl)-1H-pyrazole-4-carboxamide **(1b):** White crystals, m.p. 198-200 °C, yield (82%). IR (KBr) ν_{max}/cm^{-1} 3358, 3047 (NH, NH_2), 1640 (C=O), 1617 (C=N). ^1H-NMR (DMSO-d_6, δ ppm) 2.21 (s, 3H, CH_3), 3.64 (s, 3H, OCH_3), 5.95 (s, 2H, NH_2, D_2O exchangeable), 6.76 (d, 2H, aromatic, *J*=8.4 Hz), 7.04 (d, 2H, aromatic, *J*=8.4 Hz), 7.16 (d, 2H, aromatic, *J*=8.4 Hz), 7.33 (d, 2H, aromatic, *J*=8.4 Hz), 8.30 (s, 1H, NH, D_2O exchangeable), 8.66 (s, 1H, NH, D_2O exchangeable), 11.21 (s, 1H, NH, D_2O exchangeable). Anal. Calcd. (%) for $C_{18}H_{19}N_5O_2$ (337.38): C, 64.08; H, 5.68; N, 20.76. Found: C, 64.25; H, 5.50; N, 20.90 %.

5-Amino-3-(4-methoxyphenylamino)-N-(4-chlorophenyl)-1H-pyrazole-4-carboxamide **(1c):** White crystals, m.p. 190-192 °C, yield (80%). IR (KBr) ν_{max}/cm^{-1} 3347, 3031 (NH, NH_2), 1642 (C=O), 1613 (C=N). ^1H NMR (DMSO-d_6, δ ppm) 3.64 (s, 3H, OCH_3), 6.00 (s, 2H, NH_2, D_2O exchangeable), 6.77-7.51 (m, 8H, aromatic), 8.30 (s, 1H, NH, D_2O exchangeable), 8.82 (s, 1H, NH, D_2O exchangeable), 11.20 (s, 1H, NH, D_2O exchangeable). ^{13}C NMR (DMSO-d_6, δ ppm) 55.7 (-OCH_3), 87.9 (C_4, pyrazole), 114.7, 117.7, 121.9, 126.8, 128.9, 137.4, 138.4 (11C, aromatic), 149.1 (C_5, pyrazole), 151.1 (C_3, pyrazole), 153.2 (C, aromatic), 163.6 (C=O, amide). MS *m/z*: 358 [M^+]. Anal. Calcd. (%) for $C_{17}H_{16}N_5O_2$ (357.79): C, 57.07; H, 4.51; N, 19.57. Found: C, 57.30; H, 4.40; N, 19.75 %.

***N*-Aryl-2-[(4-methoxyphenyl)amino]-5,7-dimethylpyrazolo[1,5-*a*]-pyrimidine-3-carboxamides 5a-c:-**

2-(4-Methoxyphenylamino)-5,7-dimethyl-N-phenylpyrazolo[1,5-a]pyrimidine-3-carboxamide **(5a):** White crystals, m.p. 215-217 °C, yield (85%). IR (KBr) ν_{max}/cm^{-1} 3305 (NH), 1653 (C=O), 1599 (C=N), 1567 (C=C, aromatic). ^1H NMR (DMSO-d_6, δ ppm) 2.50 (s, 3H, CH_3), 2.56 (s, 3H, CH_3), 3.70 (s, 3H, OCH_3), 6.85 (d, 2H, aromatic, *J*=6.7 Hz), 6.89 (s, 1H, pyrimidine H-6), 7.06 (t, 1H, aromatic, *J*=7.4 Hz), 7.33 (t, 2H, aromatic, *J*=7.8 Hz), 7.59 (d, 2H, aromatic, *J*=8.8 Hz), 7.63 (d, 2H, aromatic, *J*=7.8 Hz), 9.07 (s, 1H, NH, D_2O exchangeable), 9.94 (s, 1H, NH, D_2O exchangeable). MS *m/z*: 387 [M^+]. Anal. Calcd. (%) for $C_{22}H_{21}N_5O_2$ (387.43): C, 68.20; H, 5.46; N, 18.08. Found: C, 68.28; H, 5.40; N, 18.00 %.

2-(4-Methoxyphenylamino)-5,7-dimethyl-N-(4-methylphenyl)-pyrazolo[1,5-a]pyrimidine-3-carboxamide **(5b):** White crystals, m.p. 260-261 °C, yield (88%). IR (KBr) ν_{max}/cm^{-1} 3317 (NH), 1663 (C=O), 1596 (C=N), 1565 (C=C, aromatic). ^1H NMR (DMSO-d_6, δ ppm) 2.25 (s, 3H, CH_3), 2.61 (s, 3H, CH_3), 2.69 (s, 3H, CH_3), 3.70 (s, 3H, OCH_3), 6.90 (d, 2H, aromatic, *J*=8.4 Hz), 7.00 (s, 1H, pyrimidine H-6), 7.16 (d, 2H, aromatic, *J*=8.3 Hz), 7.59 (d, 2H, aromatic, *J*=6.3 Hz), 7.66 (d, 2H, aromatic, *J*=6.3 Hz), 9.28 (s, 1H, NH, D_2O exchangeable), 10.01 (s, 1H, NH, D_2O exchangeable). MS *m/z*: 401 [M^+]. Anal. Calcd. (%) for $C_{23}H_{23}N_5O_2$ (401.46): C, 68.81; H, 5.77; N, 17.44. Found: C, 68.75; H, 5.81; N, 17.50 %.

2-(4-Methoxyphenylamino)-5,7-dimethyl-N-(4-chlorophenyl)-pyrazolo[1,5-a]pyrimidine-3-carboxamide **(5c):** White crystals, m.p. 254-256 °C, yield (83%). IR (KBr) ν_{max}/cm^{-1} 3314 (NH), 1664 (C=O), 1596 (C=N), 1566 (C=C, aromatic). ^1H-NMR (DMSO-d_6, δ ppm) 2.55 (s, 3H, CH_3), 2.62 (s, 3H, CH_3), 3.70 (s, 3H, OCH_3), 6.88 (d, 2H, aromatic, *J*=7.8 Hz), 6.92 (s, 1H, pyrimidine H-6), 7.36 (d, 2H, aromatic, *J*=7.8 Hz), 7.61 (d, 2H, aromatic, *J*=6.7 Hz), 7.68 (d, 2H, aromatic, *J*=6.7 Hz), 9.06 (s, 1H, NH, D_2O exchangeable), 10.03 (s, 1H, NH, D_2O exchangeable). ^{13}C-NMR ($CDCl_3$, δ ppm) 17.3, 24.6 (-$2CH_3$), 55.6 (-OCH_3), 86.7

(C_3, pyrazolopyrimidine), 108.5 (C_6, pyrazolopyrimidine), 114.2, 118.7, 120.5, 128 (8C, aromatic), 134.0 (C_{3a}, pyrazolopyrimidine), 137.5, 145.7, 146.0 (3C, aromatic), 154.2 (C_7, pyrazolopyrimidine), 156.8 (C, aromatic), 156.9 (C_2, pyrazolopyrimidine), 160.1 (C_5, pyrazolopyrimidine), 163.0 (C=O, amide). MS *m/z*: 421 [M^+]. Anal. Calcd. (%) for $C_{22}H_{20}ClN_5O_2$ (421.88): C, 62.63; H, 4.78; N, 16.60. Found: C, 62.70; H, 4.75; N, 16.50 %.

***N*-Aryl-5,7-dihydroxy-2-(4-methoxyphenylamino)pyrazolo[1,5-*a*]pyrimidine-3-carboxamides 9a-c:-**

5,7-Dihydroxy-2-(4-methoxyphenylamino)-N-phenylpyrazolo[1,5-a]pyrimidine-3-carboxamide **(9a):** White crystals, m.p. 208-210 °C, yield (68%). IR (KBr) ν_{max}/cm^{-1} 3375 (OH), 3303 (NH), 1650 (C=O). ^{1}H-NMR (400 MHz, DMSO-*d*$_6$): δ 3.70 (s, 3H, OCH_3), 6.80 (s, 1H, pyrimidine H-6), 6.85 (d, 2H, *J*=8.2 Hz, ArH), 7.07 (t, 1H, *J*=7.4 Hz, ArH), 7.30 (d, 2H, *J*=8.2 Hz, ArH), 7.33 (t, 2H, *J*=7.9 Hz, ArH), 7.58 (d, 2H, *J*=7.9 Hz, ArH), 8.25 (s, 1H, OH), 9.22 (s, 1H, NH), 10.35 (s, 1H, NH), 12.52 (s, 1H, OH). ^{13}C-NMR (100 MHz, DMSO-*d*$_6$): δ 55.6 (-OCH_3), 76.8 (C_6, pyrazolopyrimidine), 89.2 (C_3, pyrazolopyrimidine), 114.9, 120.2, 124.3, 128.7, 129.3 (9C, Ar), 131.9 (C_{3a}, pyrazolopyrimidine), 134.1, 138.3, 150.3 (3C, Ar), 154.1 (C_2, pyrazolopyrimidine), 160.6 (C=O), 162.1 (C_5, pyrazolopyrimidine), 162.4 (C_7, pyrazolopyrimidine). Anal. Calcd. (%) for $C_{20}H_{17}N_5O_4$ (391.38): C, 61.38; H, 4.38; N, 17.89. Found: C, 61.30; H, 4.41; N, 17.96 %.

5,7-Dihydroxy-2-(4-methoxyphenylamino)-N-(4-methylphenyl)pyrazolo[1,5-a]pyrimidine-3-carboxamide **(9b):** White crystals, m.p. 200-202 °C, yield (65%). IR (KBr) ν_{max}/cm^{-1} 3358 (OH), 3298 (NH), 1652 (C=O). ^{1}H-NMR (400 MHz, DMSO-*d*$_6$): δ 2.26 (s, 3H, CH_3), 3.70 (s, 3H, OCH_3), 6.85 (s, 1H, pyrimidine H-6), 6.84 (d, 2H, *J*=8.4 Hz, ArH), 7.13 (d, 2H, *J*=8.2 Hz, ArH), 7.28 (2H, ArH), 7.46 (d, 2H, *J*=8.2 Hz, ArH), 8.28 (s, 1H, OH), 9.12 (s, 1H, NH), 10.34 (s, 1H, NH), 12.51 (s, 1H, OH). ^{13}C-NMR (100 MHz, DMSO-*d*$_6$): δ 20.9 (-CH3), 55.6 (-OCH_3), 72.1 (C_6, pyrazolopyrimidine), 90.9 (C_3, pyrazolopyrimidine), 114.7, 117.8, 120.3, 129.5 (8C, Ar), 130.2 (C_{3a}, pyrazolopyrimidine), 136.6, 137.5, 138.3, 151.3 (4C, Ar), 153.5 (C_2, pyrazolopyrimidine), 161.8 (C=O), 162.6 (C_5, pyrazolopyrimidine), 163.0 (C_7, pyrazolopyrimidine). Anal. Calcd. (%) for $C_{21}H_{19}N_5O_4$ (405.41): C, 62.22; H, 4.72; N, 17.27. Found: C, 62.15; H, 4.75; N, 17.30 %.

5,7-Dihydroxy-2-(4-methoxyphenylamino)-N-(4-chlorophenyl)pyrazolo[1,5-a]pyrimidine-3-carboxamide **(9c):** White crystals, m.p. 165-166 °C, yield (60%). IR (KBr) ν_{max}/cm^{-1} 3347 (OH), 3239 (NH), 1659 (C=O). ^{1}H-NMR (400 MHz, DMSO-*d*$_6$): δ 3.70 (s, 3H, OCH_3), 6.76 (s, 1H, pyrimidine H-6), 6.81 (d, 2H, *J*=9.0 Hz, ArH), 7.24 (d, 2H, *J*=8.9 Hz, ArH), 7.38 (d, 2H, *J*=8.8 Hz, ArH), 7.64 (d, 2H, *J*=8.9 Hz, ArH), 9.38 (s, 1H, OH), 10.34 (s, 1H, NH), 11.26 (s, 1H, NH), 12.49 (s, 1H, OH). ^{13}C-NMR (100 MHz, DMSO-*d*$_6$): δ 55.6 (-OCH_3), 72.1 (C_6, pyrazolopyrimidine), 88.0 (C_3, pyrazolopyrimidine), 114.6, 117.7, 121.8, 128.9 (8C, Ar), 130.3 (C_{3a}, pyrazolopyrimidine), 137.2, 138.4, 149.3, 150.8 (4C, Ar), 153.2 (C_2, pyrazolopyrimidine), 161.9 (C=O), 162.7 (C_5, pyrazolopyrimidine), 163.6 (C_7, pyrazolopyrimidine). Anal. Calcd. (%) for $C_{20}H_{16}N_5O_4$ (425.83): C, 56.41; H, 3.79; N, 16.45. Found: C, 56.50; H, 3.75; N, 16.40 %.

7-Aryl-2-(arylamino)pyrazolo[1,5-*a*]pyrimidine-3-carboxamides 13a-i:-

2-(4-Methoxyphenylamino)-N,7-diphenylpyrazolo[1,5-a]pyrimidine-3-carboxamide (**13a**): Yellow crystals, m.p. 218–220 °C, yield (72%). IR (KBr) ν_{max}/cm^{-1} 3346 (NH), 1658 (C=O). ^{1}H-NMR ($CDCl_3$, 400 MHz, δ ppm): 3.80 (s, 3H, OCH_3), 6.88 (d, 2H, *J*=9.0 Hz, ArH), 6.96 (d, 1H, *J*=4.8 Hz, pyrimidine), 7.12 (t, 1H, ArH), 7.36–7.42 (m, 5H, ArH), 7.62 (d, 2H, *J*=9.0 Hz, ArH), 7.74 (d, 2H, *J*=8.4 Hz, ArH), 8.11 (d, 2H, *J*=8.3 Hz, ArH), 8.49 (d, 1H, *J*=4.8 Hz, pyrimidine), 9.40 (s, 1H, NH), 10.05 (s, 1H, NH). ^{13}C-NMR ($CDCl_3$, 100 MHz, δ ppm): 55.7 (C, OCH_3), 87.8 (C, C_3-pyrazolopyrimidine), 107.0 (C, C_6-pyrazolopyrimidine), 114.4, 119.2, 120.2, 123.7, 127.7, 129.1, 129.5, 129.6 (14C, Ar), 134.1 (C, C_{3a}-pyrazolopyrimidine), 138.8, 142.4, 146.7 (3C, Ar), 147.9 (C, C_7-pyrazolopyrimidine), 149.6 (C, Ar), 154.5 (C, C_2-pyrazolopyrimidine), 157.8 (C, C_5-pyrazolopyrimidine), 163.3 (C=O). MS (*m/z*, %): 435 (M^+, 73.86). Anal. Calcd. (%) for $C_{26}H_{21}N_5O_2$ (435.48): C, 71.71; H, 4.86; N, 16.08.Found: C, 71.80; H, 4.81; N, 16.00%.

2-(4-Methoxyphenylamino)-N-phenyl-7-(4-methylphenyl)-pyrazolo[1,5-a]pyrimidine-3-carboxamide (**13b**): Yellow crystals, m.p. 219–221 °C, yield (77%).IR (KBr) ν_{max}/cm^{-1} 3337 (NH), 1658 (C=O). ^{1}H-NMR ($CDCl_3$, 400 MHz, δ ppm): 2.49 (s, 3H, CH_3), 3.80 (s, 3H, OCH_3), 6.87 (d, 2H, *J*=8.9 Hz, ArH), 6.91 (d, 1H, *J*=4.7 Hz, pyrimidine), 7.12 (t, 1H, ArH), 7.36 (d, 2H, *J*=8.3 Hz, ArH), 7.38 (t, 2H, ArH), 7.60 (d, 2H, *J*=8.9 Hz, ArH), 7.73 (d, 2H, *J*=7.6 Hz, ArH), 8.08 (d, 2H, *J*=8.1 Hz, ArH), 8.43 (d, 1H, *J*=4.7 Hz, pyrimidine), 9.38 (s, 1H, NH), 10.01 (s, 1H, NH).^{13}C-NMR ($CDCl_3$, 100 MHz, δ ppm): 21.8 (C, CH_3), 55.7 (C, OCH_3), 87.7 (C, C_3-pyrazolopyrimidine), 107.0 (C, C_6-pyrazolopyrimidine), 114.4, 119.1, 120.1, 123.7, 127.6, 129.1, 129.4, 129.6 (14C, Ar), 134.1 (C, C_{3a}-pyrazolopyrimidine), 138.8, 142.3, 146.6 (3C, Ar), 147.8 (C, C_7-pyrazolopyrimidine), 149.6 (C, Ar), 154.4 (C, C_2-pyrazolopyrimidine), 157.7 (C, C_5-pyrazolopyrimidine), 163.3 (C=O). MS (*m*/*z*, %): 449 (M^+, 67.43). Anal. Calcd. (%) for $C_{27}H_{23}N_5O_2$ (449.50): C, 72.14; H, 5.16; N, 15.58. Found: C, 72.10; H, 5.20; N, 15.60 %.

7-(4-Chlorophenyl)-2-(4-methoxyphenylamino)-N-phenylpyrazolo[1,5-a]pyrimidine-3-carboxamide (**13c**): Yellow crystals, m.p. 252-253 °C, yield (72%). IR (KBr) ν_{max}/cm^{-1} 3343 (NH), 1648 (C=O). ^{1}H-NMR ($CDCl_3$, 400 MHz, δ ppm): 3.81 (s, 3H, OCH_3), 6.88 (d, 2H, *J*=9.0 Hz, ArH), 6.94 (d, 1H, *J*=4.7 Hz, pyrimidine), 7.13 (t, 1H, ArH), 7.39 (t, 2H, ArH), 7.58 (d, 4H, *J*=8.8 Hz, ArH), 7.74 (d, 2H, *J*=8.6 Hz, ArH), 8.15 (d, 2H, *J*=8.7 Hz, ArH), 8.52 (d, 1H, *J*=4.7 Hz, pyrimidine), 9.42 (s, 1H, NH), 9.99 (s, 1H, NH). ^{13}C-NMR ($CDCl_3$, 100 MHz, δ ppm): 55.7 (C, OCH_3), 88.0 (C, C_3-pyrazolopyrimidine), 107.0 (C, C_6-pyrazolopyrimidine), 114.4, 119.2, 120.2, 123.8, 129.1, 129.1, 130.9, 131.8 (14C, Ar), 133.9 (C, C_{3a}-pyrazolopyrimidine), 134.6, 138.0, 138.7 (3C, Ar), 145.3 (C, C_7-pyrazolopyrimidine), 149.7 (C, Ar), 154.6 (C, C_2-pyrazolopyrimidine), 157.9 (C, C_5-pyrazolopyrimidine), 163.2 (C=O). MS (*m*/*z*, %): 469 (M^+, 78.23). Anal. Calcd. (%) for $C_{26}H_{20}ClN_5O_2$ (469.92): C, 66.45; H, 4.29; N, 14.90.Found: C, 66.40; H, 4.30; N, 14.95%.

2-(4-Methoxyphenylamino)-7-phenyl-N-(4-methylphenyl)-pyrazolo[1,5-a]pyrimidine-3-carboxamide(**13d**): Yellow crystals, m.p. 251-253 °C, yield (76%). IR (KBr) ν_{max}/cm^{-1} 3374 (NH), 1660 (C=O). ^{1}H-NMR ($CDCl_3$, 400 MHz, δ ppm): 2.35 (s, 3H, CH_3), 3.80 (s, 3H, OCH_3), 6.88 (d, 2H, *J*=9.0 Hz, ArH), 6.95 (d, 1H, *J*=4.8 Hz, pyrimidine), 7.18 (d, 2H, *J*=8.2 Hz, ArH), 7.40 (d, 2H, *J*=8.2 Hz, ArH), 7.60–7.64 (m, 5H, ArH), 8.11 (d, 2H, *J*=8.2 Hz, ArH), 8.48 (d, 1H, *J*=4.8 Hz, pyrimidine), 9.42 (s, 1H, NH), 9.97 (s, 1H, NH). ^{13}C-NMR ($CDCl_3$, 100 MHz, δ ppm): 21.0 (C, CH_3), 55.7 (C, OCH_3), 87.8 (C, C_3-pyrazolopyrimidine), 106.9 (C, C_6-pyrazolopyrimidine), 114.4, 119.1, 120.2, 127.7, 129.4, 129.6, 133.3 (14C, Ar), 134.2 (C, C_{3a}-pyrazolopyrimidine), 136.2, 142.4, 146.6 (3C, Ar), 147.8 (C, C_7-pyrazolopyrimidine), 149.6 (C, Ar), 154.4 (C, C_2-pyrazolopyrimidine), 157.8 (C, C_5-pyrazolo-pyrimidine), 163.2 (C=O). MS (*m*/*z*, %): 449 (M^+, 92.11). Anal. Calcd. (%) for $C_{27}H_{23}N_5O_2$ (449.50): C, 72.14; H, 5.16; N, 15.58.Found: C, 72.20; H, 5.11; N, 15.50%.

2-(4-Methoxyphenylamino)-N,7-di-(4-methylphenyl)pyrazolo[1,5-a]pyrimidine-3-carboxamide (**13e**): Yellow crystals, m.p. 261 °C, yield (74%). IR (KBr) ν_{max}/cm^{-1} 3293 (NH), 1642 (C=O). ^{1}H-NMR ($CDCl_3$, 400 MHz, δ ppm): 2.35 (s, 3H, CH_3), 2.49 (s, 3H, CH_3), 3.80 (s, 3H, OCH_3), 6.87 (d, 2H, *J*=9.0 Hz, ArH), 6.91 (d, 1H, *J*=4.8 Hz, pyrimidine), 7.18 (d, 2H, *J*=8.2 Hz, ArH), 7.37 (d, 2H, *J*=8.0 Hz, ArH), 7.60 (d, 2H, *J*=9.0 Hz, ArH), 7.61 (d, 2H, *J*=8.5 Hz, ArH), 8.08 (d, 2H, *J*=8.3 Hz, ArH), 8.43 (d, 1H, *J*=4.8 Hz, pyrimidine), 9.40 (s, 1H, NH), 9.94 (s, 1H, NH). ^{13}C-NMR ($CDCl_3$, 100 MHz, δ ppm): 21.0 (C, CH_3), 21.8 (C, CH_3), 55.7 (C, OCH_3), 87.7 (C, C_3-pyrazolopyrimidine), 106.9 (C, C_6-pyrazolopyrimidine), 114.4, 119.1, 120.1, 127.6, 129.4, 129.5, 129.6, 133.2 (14C, Ar), 134.1 (C, C_{3a}-pyrazolopyrimidine), 136.2, 142.3, 146.5 (3C, Ar), 147.7 (C, C_7-pyrazolopyrimidine), 149.5 (C, Ar), 154.4 (C, C_2-pyrazolopyrimidine), 157.7 (C, C_5-pyrazolopyrimidine), 163.2 (C=O). MS (*m*/*z*, %): 463 (M^+, 100). Anal. Calcd. (%) for $C_{28}H_{25}N_5O_2$ (463.53): C, 72.55; H, 5.44; N, 15.11. Found: C, 72.55; H, 5.44; N, 15.11%.

7-(4-Chlorophenyl)-2-(4-methoxyphenylamino)-N-(4-methylphenyl)pyrazolo[1,5-a]pyrimidine-3-carboxamide (**13f**): Yellow crystals, m.p. 267–269 °C, yield (71%). IR (KBr) ν_{max}/cm^{-1} 3315 (NH), 1662 (C=O). ^{1}H-NMR ($CDCl_3$, 400 MHz, δ ppm): 2.35 (s, 3H, CH_3), 3.81 (s, 3H, OCH_3), 6.88 (d, 2H, *J*=9.0 Hz, ArH), 6.93 (d, 1H, *J*=4.7 Hz, pyrimidine), 7.19 (d, 2H, *J*=8.2 Hz, ArH), 7.57–7.62 (m, 6H, ArH), 8.14 (d, 2H, *J*=8.7 Hz, ArH), 8.50 (d, 1H, *J*=4.7 Hz, pyrimidine), 9.43 (s, 1H, NH), 9.91 (s, 1H, NH). ^{13}C-NMR ($CDCl_3$, 100 MHz, δ ppm): 21.0 (C, CH_3), 55.7 (C, OCH_3), 88.0 (C, C_3-pyrazolopyrimidine), 107.0 (C, C_6-

pyrazolopyrimidine), 114.4, 119.2, 120.2, 129.1, 129.6, 130.9, 131.7, 133.4 (14C, Ar), 134.1 (C, C_{3a}-pyrazolopyrimidine), 134.4, 136.0, 137.9 (3C, Ar), 146.1 (C, C_7-pyrazolopyrimidine), 149.7 (C, Ar), 154.6 (C, C_2-pyrazolopyrimidine), 159.4 (C, C_5-pyrazolopyrimidine), 163.1 (C=O). MS (*m/z*, %): 483 (M^+, 87.08). Anal. Calcd. (%) for $C_{27}H_{22}ClN_5O_2$ (483.95): C, 67.01; H, 4.58; N, 14.47. Found: C, 67.10; H, 4.50; N, 14.50%.

N-(4-Chlorophenyl)-2-(4-methoxyphenylamino)-7-phenylpyrazolo[1,5-a]pyrimidine-3-carboxamide (**13g**): Yellow crystals, m.p. 252–254 °C, yield (73%). IR (KBr) ν_{max}/cm^{-1} 3336 (NH), 1650 (C=O). ^{1}H-NMR (DMSO-d_6, 400 MHz, δ ppm): 3.72 (s, 3H, OCH_3), 6.90 (d, 2H, *J*=9.0 Hz, ArH), 7.40 (d, 1H, *J*=4.8 Hz, pyrimidine), 7.44 (d, 2H, *J*=8.8 Hz, ArH), 7.61 (d, 2H, *J*=9.0 Hz, ArH), 7.68–7.70 (m, 3H, ArH), 7.78 (d, 2H, *J*=8.9 Hz, ArH), 8.23 (d, 2H, *J*=7.2 Hz, ArH), 8.75 (d, 1H, *J*=4.8 Hz, pyrimidine), 9.20 (s, 1H, NH), 10.11 (s, 1H, NH). ^{13}C-NMR (DMSO-d_6, 100 MHz, δ ppm): 55.7 (C, OCH_3), 87.9 (C, C_3-pyrazolopyrimidine), 107.0 (C, C_6-pyrazolopyrimidine), 114.5, 119.1, 120.2, 124.0, 128.4, 129.1, 130.9, 131.8 (13C, Ar), 134.0 (C, C_{3a}-pyrazolopyrimidine), 134.6, 135.9, 138.0, 138.7 (4C, Ar), 145.6 (C, C_7-pyrazolopyrimidine), 149.7 (C, Ar), 154.8 (C, C_2-pyrazolopyrimidine), 158.0 (C, C_5-pyrazolopyrimidine), 163.8 (C=O). MS (*m/z*, %): 469 (M^+, 29.83). Anal. Calcd. (%) for $C_{26}H_{20}ClN_5O_2$ (469.92): C, 66.45; H, 4.29; N, 14.90. Found: C, 66.40; H, 4.35; N, 14.85%.

N-(4-Chlorophenyl)-2-(4-methoxyphenylamino)-7-(4-methylphenyl)pyrazolo[1,5-a]pyrimidine-3-carboxamide (**13h**): Yellow crystals, m.p. 261 °C, yield (75%). IR (KBr) ν_{max}/cm^{-1} 3322 (NH), 1658 (C=O). ^{1}H-NMR (DMSO-d_6, 400 MHz, δ ppm): 2.32 (s, 3H, CH_3), 3.74 (s, 3H, OCH_3), 6.93 (d, 2H, *J*=7.6 Hz, ArH), 7.42 (d, 1H, *J*=4.8 Hz, pyrimidine), 7.45 (d, 2H, *J*=7.7 Hz, ArH), 7.51 (d, 2H, *J*=8.6 Hz, ArH), 7.64 (d, 2H, *J*=7.8 Hz, ArH), 7.79 (d, 2H, *J*=8.4 Hz, ArH), 8.19 (d, 2H, *J*=7.5 Hz, ArH), 8.74 (d, 1H, *J*=4.8 Hz, pyrimidine), 9.23 (s, 1H, NH), 10.14 (s, 1H, NH). ^{13}C-NMR (DMSO-d_6, 100 MHz, δ ppm): 21.0 (C, CH_3), 55.7 (C, OCH_3), 87.5 (C, C_3-pyrazolopyrimidine), 107.0 (C, C_6-pyrazolopyrimidine), 114.4, 119.3, 120.9, 129.0, 129.6, 131.0, 131.7, 133.4 (14C, Ar), 134.0 (C, C_{3a}-pyrazolopyrimidine), 134.3, 136.0, 137.9 (3C, Ar), 146.1 (C, C_7-pyrazolopyrimidine), 149.7 (C, Ar), 154.5 (C, C_2-pyrazolopyrimidine), 159.4 (C, C_5-pyrazolopyrimidine), 163.1 (C=O). MS (*m/z*, %): 483 (M^+, 22.71). Anal. Calcd. (%) for $C_{27}H_{22}ClN_5O_2$ (483.95): C, 67.01; H, 4.58; N, 14.47. Found: C, 67.10; H, 4.50; N, 14.55%.

N,7-bis(4-Chlorophenyl)-2-(4-methoxyphenylamino)pyrazolo[1,5-a]pyrimidine-3-carboxamide (**13i**): Yellow crystals, m.p. 282–284 °C, yield (70%). IR (KBr) ν_{max}/cm^{-1} 3317 (NH), 1653 (C=O). ^{1}H-NMR (DMSO-d_6, 400 MHz, δ ppm): 3.74 (s, 3H, OCH_3), 6.94 (d, 2H, *J*=8.8 Hz, ArH), 7.44 (d, 2H, *J*=8.6 Hz, ArH), 7.45 (d, 1H, *J*=3.8 Hz, pyrimidine), 7.61 (d, 2H, *J*=8.8 Hz, ArH), 7.78 (d, 4H, *J*=8.4 Hz, ArH), 8.29 (d, 2H, *J*=8.6 Hz, ArH), 8.76 (d, 1H, *J*=4.7 Hz, pyrimidine), 9.23 (s, 1H, NH), 10.10 (s, 1H, NH). ^{13}C-NMR (DMSO-d_6, 100 MHz, δ ppm): 55.8 (C, OCH_3), 87.4 (C, C_3-pyrazolopyrimidine), 106.9 (C, C_6-pyrazolopyrimidine), 115.0, 119.3, 120.4, 129.1, 129.7, 129.9, 131.6 (13C, Ar), 133.1 (C, C_{3a}-pyrazolopyrimidine), 133.8, 134.3, 136.0, 137.9 (4C, Ar), 146.0 (C, C_7-pyrazolopyrimidine), 149.8 (C, Ar), 154.5 (C, C_2-pyrazolopyrimidine), 159.4 (C, C_5-pyrazolopyrimidine), 162.9 (C=O). MS (*m/z*, %): 504 (M^+, 22.87). Anal. Calcd. (%) for $C_{26}H_{19}Cl_2N_5O_2$ (504.37): C, 61.91; H, 3.80; N, 13.89. Found: C, 62.00; H, 3.75; N, 13.80%.

In Vitro Biological Evaluation

Test Microorganisms

The test microorganisms were used in this study, Gram-positive bacteria: Bacillus cereus (ATCC14579, Bc), Staphylococcus aureus (ATCC 29213, Sa) and Enterococcus faecalis (ATCC 29212, Ef). Gram-negative bacteria: Escherichia coli (ATCC 25922, Ec), Pseudomonas aeruginosa (ATCC 27853, Pa) and Salmonella typhi (ATCC 6539, St). Fungi: Candida albicans (ATCC 10231, Ca), Fusarium oxysporum (RCMB 008002, Fo) and Aspergillus brasiliensis (ATCC 16404, Ab).

Antimicrobial Activity

The antimicrobial activities inhibition zone (IZ, mm±standard deviation) was measurement according to the agar plate diffusion method. Briefly, 100 μl of the test bacteria/fungi were grown in 10 mL of fresh media until they reached a count of approximately 108 cells/ml for bacteria or 105 cells/mL for fungi. One mL of each sample (at 0.5 mg/mL) was added to each well (10 mm diameter holes cut in the agar gel). The plates were incubated for 24 h at 37 ^{o}C (for bacteria and yeast) and for 72 h at 27 ^{o}C (for filamentous fungi), each test was repeated three times. After incubation, the microorganism's growth was observed. Tetracycline was used as standard antibacterial drugs while amphotericin B was used as standard antifungal drug. The resulting inhibition zone diameters were measured in millimeters and used as criterion for the antimicrobial activity. Solvent controls (DMSO) were included in every experiment as negative controls. DMSO was used for dissolving the tested compounds and showed no inhibition zones, confirming that it has no influence on growth of the tested microorganisms.

The MIC, MBC and MFC of the potent pyrazolo[1,5-a]pyrimidines:

The minimum inhibitory concentration (MIC), the minimum bactericidal concentration (MBC, μg/ml) and the minimum fungicidal concentration (MFC, μg/ml) of the most potent pyrazolo[1,5-*a*]pyrimidines {**5c**, **9a**, **9c**, **13a**, **13c**, **13d**, **13e** and **13h**} were determined by the conventional technique termed paper disk diffusion, by applying paper disk (266812 W. Germany 12.7 mm in diameters). Bacteria were grown on nutrient agar medium, while fungi and yeast were grown on Sabouraud agar medium. The purified streptomycin was dissolved in water and loaded on paper disks with different concentrations as the following (250, 125, 62.50, 31.25, 15.63, 7.81, 3.90, 1.95, 0.98, 0.49, 0.24 and 0.12 μg/mL). Drying disks were loaded on surface of agar plates inoculated with test organism. Growth inhibition was examined after 24 hr. from incubation at 37 ^{o}C for bacteria and after 72 hr. incubation at 27 ^{o}C for fungi and yeast. Each test was repeated three times. MIC was expressed as the lowest concentration inhibiting test organism's growth.

Immunomodulatory activity of the potent pyrazolo[1,5-a]pyrimidines compounds:-

Isolation of neutrophils

A 15 mL peripheral blood sample was taken from healthy volunteers collected in preservative-free heparin and 2 mL of 4.5% dextran B in saline was added. After gently shaking, the mixture was allowed to stand for 60 min at 37 °C to sediment erythrocytes. Neutrophils were isolated by centrifugation. After removal of the residual erythrocytes by hypotonic lysis the neutrophils were washed with Hank's balanced salt solution (HBSS) (Sigma, St Louis, USA) and then suspended at a final concentration of 25×10^{6} cells/mL in HBSS for intracellular killing activity. The viability of neutrophils was tested by trypan blue exclusion and was greater than 90%.

Intracellular killing activity

The intracellular killing activity or the respiratory burst of neutrophils was measured by nitroblue tetrazolium (NBT) reduction test by the modified method**.** Isolated neutrophils were incubated in HBSS with latex particles, NBT (Sigma, St Louis, USA) and the isolated flavonoids in the same concentrations as used in chemotaxis for 30 min at 37°C, then the reduced dye, blue formazon was extracted with pyridine and measured spectrophotometrically at 515 nm. The results were compared with the negative control consisting all the reagents except the neutrophil suspension. The difference in the absorbance between the cultures of cells that actively phagocyte latex particles and the negative control was considered as an index of intracellular killing activity of neutrophils. Activity of the samples was calculated as the percentage ratio of the compounds in latex to positive control which is latex.
